# Retraction Note: *Sirt7* promotes gastric cancer growth and inhibits apoptosis by epigenetically inhibiting miR-34a

**DOI:** 10.1038/s41598-023-37359-8

**Published:** 2023-06-26

**Authors:** Shun Zhang, Ping Chen, Zuoan Huang, Xiaorong Hu, Mengting Chen, Shanshan Hu, Yixin Hu, Ting Cai

**Affiliations:** 1 Stem Cell Laboratory, Ningbo No.2 Hospital, Ningbo, 315010 Zhejiang China; 2 Gastrointestinal and Hernia Ward, Ningbo No.2 Hospital, Ningbo, 315010 Zhejiang China; 3 Department of Internal Medicine, School of Medicine, Ningbo University, Ningbo, 315211 Zhejiang China

Retraction of: *Scientific Reports* 10.1038/srep09787, published online 10 April 2015

The Editors have retracted this Article.

After the publication, the first author reached out to the Editors to point out that that the phase contrast microscopy image in Figure 3(b) appears to replicate panels that originally appeared in Figure 2(b) of^1^, which reports a different experiment by different authors. When asked by the Editors, the Authors were not able to provide the raw data and stated that the images in Figure 3(b) were obtained through a third party hired to perform some of the experiments for this study. Given the concerns about the veracity of the data, the Editors no longer have confidence in the results and conclusions presented in this Article.

All Authors agree to this retraction.
